# *Daniellia oliveri* (Rolfe) Hutch and Dalziel: Antimicrobial Activities, Cytotoxicity Evaluation, and Phytochemical Identification by GC-MS

**DOI:** 10.3390/antibiotics11121699

**Published:** 2022-11-25

**Authors:** Nassifatou Koko Tittikpina, Gilbert Kirsch, Raphaël Emmanuel Duval, Patrick Chaimbault, Claus Jacob

**Affiliations:** 1 Department of Bioorganic Chemistry, School of Pharmacy, University of Saarland, Campus B2 1, D-66123 Saarbruecken, Germany; 2 LCP-A2MC, Université de Lorraine, F-57000 Metz, France; 3 CNRS, L2CM, Université de Lorraine, F-57000 Metz, France; 4 CNRS, L2CM, Université de Lorraine, F-54000 Nancy, France; 5 ABC Platform^®^, Faculté de Pharmacie, F-54001 Nancy, France; 6 Département des Sciences Pharmaceutiques, Faculté des Sciences de la Santé, Université de Lomé, Lomé 01 BP 1515, Togo

**Keywords:** *Daniellia oliveri*, antibacterial activity, cytotoxicity activity, phytochemical identification

## Abstract

During a previous study that identified plants used in traditional medicine in Togo to treat infectious diseases, *Daniellia oliveri* was specifically reported to treat intertrigo and candidiasis. Consequently, to explore the anti-infective potential of this plant, we investigated the antibacterial and the antifungal activity of the plant’s parts, as well as the cytotoxic activities of raw extracts and subsequent fractions, and the chemical composition of the most active fractions. In order to evaluate the antimicrobial activity, MICs were determined using the broth dilution method. Then, the most active fractions were evaluated for cytotoxicity by using normal human cells (MRC-5 cells) via the MTT assay. Finally, the most active and not toxic fractions were phytochemically investigated by GC-MS. Interestingly, all the raw extracts and fractions were active against the bacteria tested, with MICs ranging from 16 µg/mL to 256 µg/mL, while no antifungal activity was observed at 256 µg/mL, the highest tested concentration. Moreover, no toxicity was observed with most of the active fractions. The subsequent chemical investigation of the most interesting fractions led to identifying terpenes, phytosterols, phenolic compounds, and fatty acids as the main compounds. In conclusion, this study demonstrated that *D. oliveri* possesses valuable antibacterial activities in accordance with traditional use.

## 1. Introduction

In 2010, the World Health Organization (WHO) estimated that more than 80% of the people in the developing world use traditional medicine for their healthcare needs [1]. Most of those countries are in Africa. At the same time, Africa is home to great biodiversity from which traditional medicine draws herbal and medicinal plants. Such plants are indeed the main material employed in traditional medicine, and plants are administrated to patients in different forms called herbal products [2]. In addition, in the whole world, it is estimated that 25% of all modern medicine is directly or indirectly coming from higher plants [3]. Several studies have been performed all over Africa to document plants used in traditional medicine. Researchers have constantly been warning about the ever-increasing burden of infectious diseases on the healthcare policies of the continent. Such diseases have been receiving considerable interest due to the inefficacy of currently available antibiotics, lack of supply of new antibiotics, and especially due to the emergence of resistance towards conventional antibiotics [4]. Sub-Saharan Africa is the most affected part of the continent and is often referred to as the ‘Infectious continent’ [5]. Louw and colleagues have reported the significance of bulbous plants used in South Africa in traditional medicine to treat infectious diseases [6]. Other studies have documented plants used all over the continent with antibacterial, antifungal, and antiviral activities [7,8,9,10]. Other researchers went further and studied the antibacterial activity of 83 polar and non-polar extracts from 22 medicinal plants against Gram-positive (*Staphylococcus aureus* and *Enterococcus faecalis*) and Gram-negative bacteria (*Escherichia coli*, *Pseudomonas aeruginosa*, *Klebsiella pneumoniae*) and *Mycobacterium smegmatis* [11]. Africa is a treasure chest comprising several thousand, if not millions, of such medicinal plants; unfortunately, such screening could not be performed on the entire flora of Africa, and therefore certain narrowing to the most promising plants is needed. In this context, Pillay and colleagues have established a prioritization process employing selection criteria on plants used to treat certain infectious diseases in South Africa. The prioritization process led to highly ranked plants that were further investigated biologically [12]. Following their footsteps, we have also developed a model to identify the most promising plants from a list of species reported after an ethnobotanical survey for possible treatment of infectious diseases in Togo [13]. The ethnobotanical study involving a semi-structured individual interview with 53 traditional healers from the Tchamba district of Togo led to identifying 43 different species effective against infectious diseases [13,14]. This methodology, named CAPITURE (Computer Aided Products Identification from Traditional Usage Records) method, predicted *Pterocarpus erinaceus* and *Daniellia oliveri* as the most promising plants out of 43 plant species employed by traditional healers to treat infectious diseases [13].

We have previously investigated and published the studies performed on *P. erinaceus,* and as predicted, promising antibacterial activities to have been observed with this plant [14]. We are here reporting the results of the studies on *D. oliveri*. *D. oliveri* has been employed by traditional healers to treat intertrigo and candidiasis [13]. Intertrigo is caused by bacterial and fungal species, including *Pseudomonas aeruginosa*, *Staphylococcus aureus,* and *Candida albicans* [15]. Consequently, the antimicrobial activities of different parts of this plant, raw extracts, and subsequent fractions have been evaluated against a broad range of bacteria and fungi. The cytotoxic activities of the most active fractions have also been performed, in vitro, on non-cancerous human cells, and such activities have been compared with the raw extracts they are derived from. Finally, the most active non-toxic fractions were evaluated for phytochemical composition to provide a general overview of the type of phytochemicals present in the extracts.

## 2. Results

### 2.1. Antibacterial and Antifungal Activities

All the methanolic extracts (raw extracts) of the leaves, trunk barks, and roots provided antimicrobial activities against *Enterococcus faecalis* and *Staphylococcus aureus* with MICs ranging from 16 µg/mL to 256 µg/mL (Table 1). The highest activity was provided by the trunk bark extract with the lowest MIC value of 16 µg/mL against *E. faecalis*. It was also the only extract that provided antimicrobial activity against *Pseudomonas aeruginosa* and *Escherichia coli,* with a MIC value of 256 µg/mL (Table 1). No MIC was observed with the trunk bark extract on the rest of the bacteria (*Klebsiella pneumoniae*, *Enterobacter cloacae,* and *Acinetobacter baumannii*). However, the trunk bark extract inhibited the growth of those bacteria at the highest concentration of 256 µg/mL, with percentages of inhibition (PIs) varying from 75% to 86%. The raw extracts of leaves and roots presented MICs only against *Enterococcus faecalis* and *Staphylococcus aureus,* with values ranging from 32 µg/mL to 256 µg/mL. However, with the two extracts, inhibition of bacterial growth has also been recorded against the other bacteria, with PIs ranging from 37% to 88%. Anyway, the leaf extract was more potent in inhibiting bacterial growth, with PIs ranging from 74% to 88%. Then the roots’ extracts, whose PIs ranged from 37% to 59% (Table 1). The raw extracts were also tested on *Candida albicans,* and no activity was observed at the highest tested concentration (i.e., 256 µg/mL, Table 1).

**Figure 1:**
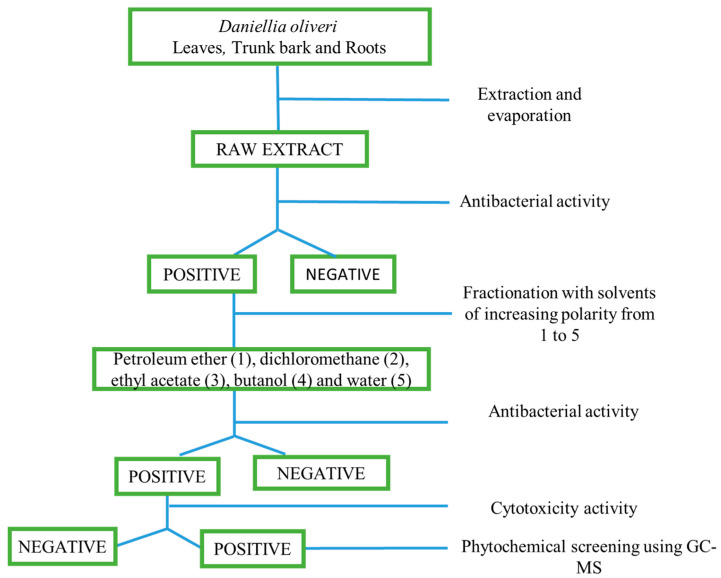
Flowchart of the bio-guided isolation assay performed on the different parts of *D. oliveri.* Raw extract: methanolic (MeOH) raw extract. Bioassays (i.e., antibacterial activity)—Positive: antibacterial activity against bacteria; Negative: no antibacterial activity. Cytotoxic activities—Positive: not toxic to normal human MRC-5 cells; Negative: toxic to MRC-5 cells.

Based on those results, we could conclude that all the raw extracts from the different parts of *D. oliveri* inhibited the growth of all seven bacteria involved in the study. Consequently, following the methodology presented in Figure 1 (materials and methods), the raw extracts were subjected to fractionation with solvents of increasing polarities: petroleum ether, dichloromethane, ethyl acetate, butanol (except for the roots), and water. Then, the obtained fractions also went through the same antibacterial tests.

As a result of the antibacterial activities of fractions, we have noticed that all the fractions were active against bacterial strains, with either MICs or PIs. 18 MICs were observed with the leaves’ fractions, 21 MICs with that of the trunk barks, and 8 MICs with the roots fraction with concentrations as low as 32 µg/mL (Table 2). Indeed, the highest antimicrobial activities were recorded for the root’s petroleum ether and dichloromethane fractions which presented the lowest MIC at 32 µg/mL against *E. faecalis*. In addition, it is also noteworthy that all the fractions of the trunk bark exhibited MICs (ranging from 64 to 256 µg/mL) against the Gram-positive bacteria *E. faecalis* and *S. aureus*. Anyway, all the fractions coming from the leaves, trunk bark, and roots were active against the same Gram-positive bacteria. For most of them, whenever a MIC was not observed on the bacteria, a percentage of inhibition was recorded with values ranging from 15% to 90% at the highest concentration tested (256 µg/mL). Anyway, the butanol fraction of the leaves was the only fraction that presented a MIC against all the bacterial strains tested. The observation was similarly made for the butanol fraction of the trunk bark, which has also shown MIC against most of the bacterial strains except against Gram-negative *E. coli* and *K. pneumoniae*. However, against those two Gram-negative bacteria, high PIs (respectively at 82.5% and 72.5%) have been recorded with the butanol fraction of the trunk bark. Another remarkable observation was that all the ethyl acetate fractions were active against the bacterial strains, showing either MICs or PIs (Table 2). In summary, we have observed MICs with the following fractions: the petroleum ether, dichloromethane, ethyl acetate, butanol, and water fractions of the trunk bark; the petroleum ether, butanol, and water fractions of the leaves and the dichloromethane, ethyl acetate and remaining methanol fraction of the roots. Because of the prevalence of cutaneous infections due to *S. aureus* in Togo [16], the most active fractions were tested against two other strains of *Staphylococcus*: methicillin-resistant *S. aureus* (MRSA) and *S. epidermidis* (Table 3).

A MIC was recorded with almost all fractions on the two additional bacteria, except with the petroleum ether fraction of the leaves, the ethyl acetate fraction of the roots, the remaining methanolic fraction of the roots on *S. epidermidis,* and the dichloromethane fraction of the trunk bark against the MRSA (a PI at 63.54% was however recorded) (Table 3). More remarkably, an inhibition of 50% of bacterial growth (PI_50_) has also been observed with the butanol and water fractions of the leaves and trunk barks against MRSA and *S. epidermidis* at a concentration as low as 1 µg/mL. The most active fractions against MRSA and *S. epidermidis* include the butanol and water fractions of the leaves, all the fractions of the trunk bark, and the dichloromethane fraction of the roots. Anyway, the remaining methanolic fraction of the roots were only active against the MRSA.

### 2.2. Cytotoxicity Activities

Since most of the fractions provided excellent antimicrobial activities, it was important to investigate their safety profile, therefore, toxicity tests were performed on the most active fractions and compared to the toxicity tests of the original raw extracts. In general, no toxicity was observed with the most active raw extracts and fractions at concentrations where they presented antimicrobial activities, except the butanol and water fractions of the trunk barks. Butanol and water fractions provided toxicity with IC_50_ of 81.18 µg/mL and 76.96 µg/mL, respectively (Table 4). With those fractions, MIC ranging from 64 µg/mL to 256 µg/mL was recorded. The values of toxicity on normal human cells, when compared to MICs values, make the toxicity not negligible (Table 4). Consequently, further investigations are needed to explore the toxicity of these fractions.

### 2.3. Phytochemical Investigation

In general, for all the non-polar extracts investigated, a chromatogram was obtained, which could be roughly subdivided into three parts: phenolic compounds from 3 to 8 min, fatty acids from 8 to 13 min, and pentacyclic triterpenes starting from 13 min onwards. The identification was confirmed through the library at more than 80% accuracy most of the time. Some compounds have also been identified with just 70% of equivalence, where some doubt could persist. This could be explained by the coelution of other metabolites with those compounds or the fact that the concentration of the compound is too low to allow unequivocal identification of fragments.

Concerning the roots, different types of terpenes were identified in the dichloromethane fraction, including a hydrocarbon sesquiterpene named δ-cadinene and a diterpene named daniellic acid. In addition, some peaks observed remained unidentified and might corresponding to never described nature compounds (Table 5).

Various compounds were also found in the trunk barks. Indeed, compounds such as fatty acids, terpenes including pentacyclic ones, phytosterols, and phenolic compounds, were deciphered in the petroleum ether (PE) and dichloromethane (DCM, after sylilation) fractions (Table 6 and Table 7). Among fatty acids, the following saturated fatty acids have been unveiled: palmitic acid in the Petroleum Ether (PE) fraction and succinic acid, myristic acid, palmitic acid (methyl ester), pentadecanoic acid, heptadecanoic acid, stearic acid, arachidic acid, behenic acid, lignoceric acid, pentacosanoic acid (hyenic acid), hexacosanoic acid (cerotic acid) in the DCM fraction (Table 6 and Table 7). In addition to saturated fatty acids, unsaturated fatty acids, such as linoleic and oleic acids were identified in the PE fraction (Table 6). Linoleic acid; fumaric acid co-eluted with an unnamed acid, 6-octadecenoic acid; 9,12-octadecadienoic acid; stearic acid; octadecenoic acid (oleic acid); gadoleic acid were identified in the DCM extract (Table 6). Anyway, some terpenoids have been deciphered in the PE fraction but were not formally named. They might corresponding to some never described terpenoids (Table 6).

Pentacyclic triterpenes, such as *β*-amyrinin and lupeol, were also identified in the DCM fraction (Table 7). Interestingly, phytosterols were discovered in the two fractions. Stigmasterol and *γ* or *β* -sitosterol were identified in the PE fraction, whilst campesterol, stigmasterol, sitosterone, and *γ* or *β* -sitosterol were found in the DCM fraction. Moreover, the DCM fraction revealed monoglycerides, namely glycerol and some unidentified isomers of monoolein and mono palmitin (Table 6 and Table 7). In addition, the following phenolic compounds, including polyphenols (flavonoids), have also been unveiled: vanillin, benzoic acid, and its derivatives, including *p*-tyrosol, protocatechuic acid, ferulic acid, syringic acid, epitachin and catechin in the DCM fraction (Table 7) with mellein in the PE fraction (Table 6).

Finally, since the leaves of *D. oliveri* were already extensively scrutinized by various researchers for phytochemicals, we did not investigate them in the present research. To sum up, the phytochemical investigation of the most active extracts of *D. oliveri* during this work has led to identifying of various phytochemicals: fatty acids, terpenes, phenolic compounds, monoglycerides, etc. Such compounds are commonly encountered in the plant kingdom and might be the ones responsible for the antibacterial activities associated with the fractions of the roots, trunk barks, and leaves fractions of *D. oliveri*. Indeed, fatty acids, terpenes, sterols, and flavonoids are largely documented to exhibit antibacterial activities, and the compounds identified therein could explain the activities associated with the fractions of the leaves, trunk barks, and roots of this plant [14,17].

## 3. Discussion

### 3.1. Antibacterial, Antifungal, and Cytotoxicity Activities

The antibacterial activities observed with the raw extract and fractions coming from the different parts of *D. oliveri* were confirmed by some studies performed on just some parts of the plant and from different locations in Africa. Indeed, to the best of our knowledge, our study is the first reporting activities on all three parts of the plants, with raw extracts and fractions and using a more sensitive method for antimicrobial testing, the broth dilution method. For example, Ahmadu and colleagues investigated the antibacterial activity of the aqueous ethanolic extract of the leaves of *D. oliveri* using the agar well diffusion assay [18,19]. Results coming from their work have shown the methanolic raw extract of the leaves to present a higher activity against *S. aureus* in comparison to that of *P. aeruginosa* and *E. coli* at concentrations ranging from 5 mg/mL to 50 mg/mL. In addition, the butanol fraction was also the most active of the leaves’ fractions [18,19]. Other researchers have also investigated the activity of the leaves and trunk barks employing the same method as in the previous study. For example, in one of those studies, the ethanolic extract of the leaves and trunk barks of *D. oliveri* inhibited bacterial growth at 60 mg/mL [20]. El-Mahmood and colleagues have also evaluated the antibacterial activities of *D. oliveri* leaves, barks, and roots ethanolic and aqueous extracts. MIC values ranging from 6.25 mg/mL to 100 mg/mL were obtained with the extracts against *E. coli*, *S. aureus*, *K. pneumoniae,* and *Shigella dysenteriae* [21]. It should be noted that, compared to the results described by earlier researchers, the antibacterial activities that we measured are much better (i.e., 100 to 1000 times) since we obtain MICs in the µg/mL range, whereas those obtained in the studies mentioned above are in the mg/mL range. The difference observed with those different studies could be explained either by a difference in the composition of the plant’s extracts since plants growing in different geographical zones tend to develop different phytochemicals and in different concentrations [22] and or by the fact that we did not use the same technique for the evaluation of the antimicrobial activity.

Unfortunately, no activity was observed with the raw extracts of *D. oliveri* against *Candida albicans,* even at the highest tested concentration (i.e., 256 µg/mL). However, the literature reveals the efficacy of the methanol extract of the leaves and the methanolic and aqueous extracts of the trunk barks against *Aspergillus niger, Candida albicans, Candida krusei, Rhizopus stolonifer, Epidermophyton floccosum, Trichophyton floccosum, Trichophyton interdigitale, and Trichophyton rubrum* with MICs ranging from 3.125 mg/mL to 200 mg/mL [23]. The difference observed with the MIC could be explained by the difference in the antifungal assays (broth dilution in the present study and agar well-diffusion in the study reported in the literature) and the place of harvest (Togo in the present study and Nigeria in the one reported in the literature). Anyway, in our study, we have tested the antifungal activity at a very low concentration (256 µg/mL) compared to the work of Coker and Ogundele in 2016 [23], where anti-fungal activity was recorded at a concentration in the mg/mL range (3.126 mg/mL to 200 mg/mL).

Concerning the toxicity profile recorded, compared to our literature search, to the best of our knowledge, no data have been reported on the toxicity of *D. oliveri* leaves and root extracts. However, the trunk bark toxicity was evaluated orally in mice in a study. The results have provided an LD_50_ (Lethal Dose that kills 50% of animals involved in the study) greater than 3.5 g/Kg in mice [24]. From the observations made from our toxicity work and from our literature review, we concluded that the leaves with petroleum, butanol, and water fractions; the trunk bark with its petroleum ether and dichloromethane fractions and the roots with dichloromethane fraction, which are not toxic; were worthy of chemical investigation. Because most fractions were non-polar fractions, we have conducted a thorough chemical analysis to provide an outline of the phytochemicals present in the extracts, which could be responsible for the activities observed, using gas chromatography coupled to mass spectrometry.

### 3.2. Phytochemical Investigation

δ-Cadinene and daniellic acid identified in the roots have also been reported in the same plant or other plants in the literature. For example, polyalthic or daniellic acid was isolated from the oleoresin of *D. oliveri* (Fabaceae) [25], whilst δ-Cadinene was reported in the roots of *Kadsura oblongifolia* (Schisandraceae) [26]. Concerning the trunk barks, the detected compounds in the extracts have either been already identified in the same plant by other authors or in other plants as reported by other studies. The present study is the first one to report the presence of several molecules in this plant. For example, our work is the first to report isocoumarins such as mellein and flavonoids such as catechin and epicatechin in this plant (to the best of our knowledge). Some authors have also recognized other coumarins and flavonoids. Indeed, compounds such as p-coumaric acid, coumarin, homo-orientin, rutin, quercitrin-glucosyl, quercitrin-dehydrate, delphinidin have been isolated or quantified from in the methanolic and aqueous fractions of the trunk barks [27,28]. Anyway, the compound mellein was reportedly isolated from the leaves and stems barks of *Stevia lucida Lagasca* (Asteraceae) [29]. In addition, flavonoids, phytosterols, terpenes, and fatty acids are also very common in the plant kingdom, and their presence in the bark of *D. oliveri* is not a surprise. Furthermore, the sterols revealed in our studies, such as β-sitosterol, stigmasterol, and campesterol, have been isolated from the stems barks of a plant named *Annona vepretorum* (Annonaceae) [30] whilst pentacyclic triterpenes identified in our study were also identified in the leaves, barks, and roots of various plants [31]. In addition, acids revealed during our analysis of *D. oliveri* fractions, such as pentadecanoic, heptadecanoic, octadecadienoic, and octadecenoic acids, were also identified in the tuber of *Lepidium meyenii* (Brasicacceae) [32] and, hexacosanoic (serotic acid) has been characterized in the peanut seed oil [33]. Anyway, monoglycerides, resulting from the esterification of palmitic acid, glycerol, and other compounds, were also unveiled in the trunk barks extract of *D. oliveri* during our analysis by GC-MS. Finally, several interesting peaks were also observed on the chromatogram but did not hint at already described or known compounds from the chemical library. Hence, those compounds might corresponding to never describe structures. Nevertheless, our analysis is the first to provide a detailed identification of non-polar compounds existing in the fractions of the trunk bark and roots of *D. oliveri* (to the best of our knowledge).

In addition, since the leaves of *D. oliveri* were already extensively scrutinized by various researchers for phytochemicals, we did not investigate them in the present research. Indeed, when investigating the antimicrobial activities of the leaves of *D. oliveri* against bacteria and fungi, researchers found the n-butanol fraction to be the most effective one. The subsequent exploration of the butan-1-ol fraction led to identifying the following flavonoids: rutin, quercitin-3/-O-methyl-3-O-α-rhamnopyranosyl-β-D-glucopyranoside, quercitrin and quercimeritrin [18,19]. Muanda and colleagues have also identified a certain number of phenolic compounds in the methanolic and aqueous extracts of the leaves, including gallic acid, procatechuic acid, catechin, chlorogenic acid, caffeic acid, p-coumaric acid and homo-orientin [29].

Finally, it is important to point out that chemical compounds, namely fatty acids, terpenes, sterols, and phenolic compounds, profiled in *Daniellia oliveri*’s extracts and fractions, are reported to possess antimicrobial activities [14,34,35,36,37,38]. Furthermore, it is noteworthy to point out that other researchers have studied the mechanisms of actions of some terpenes, fatty acids, sterols, and phenolic compounds [35,36,37,38,39,40]. For example, β-sitosterol (representative of phytosterols), identified in the barks of *D. oliveri* slows bacterial growth, leading to a static bacterial effect [35]. Pentacylic triterpenes, such as α-Amyrin (precursor of ursolic acid) and β-amyrin (precursor of oleanolic acid, identified in the barks of *D. oliveri*), inhibit the growth of bacteria. Their target is the membrane of bacteria cells, where they: disrupt ion exchanges happening through the membrane, interrupt the synthesis of proteins, and deregulate the machinery of the bacteria ribosomes [37]. The antibacterial activity of saturated fatty acids, namely palmitic and stearic acids (identified in barks of *D. oliveri*) is exerted through the inhibition of quorum sensing in bacteria, leading to changes in gene expression, biofilm synthesis, and virulence factors against immune response development. For the unsaturated fatty acids identified in *D. oliveri*, namely linoleic acid, the effect observed on Gram-positive bacteria is due to membrane cell biosynthesis inhibition [40].

## 4. Materials and Methods

### 4.1. Materials

#### 4.1.1. Plant Materials

The National Authority of Species Protection (Direction Nationale de la Protection des Végétaux, Lomé, Togo) provided a permit to collect and travel overseas with different plant parts. The plant, and its organs have been collected in the central region of Togo (GPS coordinates: 09°11′689″ North, 001°15′942″ East). *D. oliveri* has then been identified by a botanist, and a voucher specimen, the number TOGO 15076, has been deposited at the Herbarium of the University of Lomé, Togo. After collection, leaves, barks, and roots were dried at 25 °C at the laboratory of Botany and Plants Ecology and milled with a simple commercial grinder. After milling, the pulverized materials were sealed and brought to Europe by plane.

#### 4.1.2. Chemicals

Sodium dodecyl sulfate (SDS, L5750) and thiazolyl blue tetrazolium bromide (MTT, M2128) were purchased from Sigma-Aldrich (Saint-Quentin Fallavier, France). Phosphate Buffered Saline (PBS) was prepared (8 g/L NaCl, 0.2 g/L KCl, 2.9 g/L Na_2_HPO_4_, 12H_2_O, 0.2 g/L KH_2_PO_4_) and sterilized using autoclave before use.

#### 4.1.3. Bacterial and Fungal Strains

The bacterial strains involved in this study include *Enterococcus faecalis* (ATCC 29212), *Staphylococcus aureus* (ATCC 29213), *Escherichia coli* (ATCC 25922), *Klebsiella pneumoniae* (ABC 42), *Enterobacter cloacae* (ABC 291), *Pseudomonas aeruginosa* (ATCC 27853) and *Acinetobacter baumannii* (ABC 14). The most effective fractions were screened against Methicillin-Resistant *S. aureus* (MRSA, ABC 61) and *S. epidermidis* (ABC 112). The anti-fungal studies were performed against *Candida albicans*.

All strains were kindly provided by the ABC Platform^®^ Bugs Bank. All bacterial strains were cultured on Mueller Hinton Agar (MHA, Difco 225250), and the antibacterial tests were realized on Mueller Hinton Broth (MHB, Difco, 275730). The fungal strain was cultured on Sabouraud agar, and the antifungal test was realized with Roswell Park Memorial Institute (RPMI)-1640 medium without carbonates and with glutamine (Gibco, 11875101).

Bacterial and fungal strain susceptibility testing was performed during the time course experiments, using clinical reference agents (i.e., antibiotics): amoxicillin, ampicillin, oxacillin, penicillin G, ticarcillin, vancomycin, and Amphothericin B.

#### 4.1.4. Eukaryotic Cell

For the cytotoxic assays, MRC-5 cells (ATCC CCL-171, human lung fibroblasts) were used. Cells were cultured in Minimum Essential Medium (MEM, 31095-029, Life Technologies-Gibco^R^) supplemented with 10% heat-inactivated fetal calf serum (CVFSV00-0U, Eurobio, Courtaboeuf, France) and 2 mM L-glutamine (G7513-100 mL, Sigma Aldrich, Saint Louis, MD, USA), at 37 °C in a 5% CO_2_ humidified atmosphere.

### 4.2. Methods

#### 4.2.1. Extraction and Fractionation

A preliminary check with different mixtures of solvents and TLC (TLC silica gel 60 F254, Merck Millipore, 1.05554.0001) was performed to evaluate which conditions provide better separation. The best results were obtained with MeOH (100%). Consequently, 3000 g of powdered dried leaves, barks, and stems barks have been macerated at room temperature for 48 h into 3000 mL of methanol to obtain methanolic raw extracts. The supernatant from the maceration has been collected and evaporated under pressure using a rotary evaporator. The methanolic raw extracts have further been fractionated using liquid-liquid partitioning with solvents of increasing polarities: petroleum ether, dichloromethane, ethyl acetate, butanol, and distilled water (Figure 1).

The methanolic extract of the roots was not soluble in water, consequently, only petroleum ether, dichloromethane, ethyl acetate, and the remaining methanolic extract were obtained for this plant organ. The extraction and fractionation processes yield different weights of extracts (Table 8). All the solvents used are pure solvents for synthesis [14].

#### 4.2.2. Antibacterial and Antifungal Tests

The antibacterial and antifungal activities of the raw extracts and fractions were evaluated as previously reported [14,41,42] in terms of Minimal Inhibitory Concentration (MIC, the concentration that inhibits 100% of bacterial growth) using the broth dilution method and following the recommendations of the Clinical and Laboratory Standards Institute [43,44,45]. MICs, Percentage of Inhibition (PI, inhibition of bacterial or fungal growth observed at the highest concentration tested 256 µg/mL), and PI 50 (concentration of extract or fraction that inhibits 50% of bacterial or fungal growth) were determined as previously described [14,41,42,43,44,45]. The negative control wells are made of broth only or broth with extract or compound without inoculum. Vancomycin, Penicillin G, Ticarcillin, Amoxicillin, and Ceftazidime-avibactam were used as a positive control against *Enterococcus faecalis*, *Staphylococcus aureus*, *Pseudomonas aeruginosa*, *Acinetobacter baumannii*, *Escherichia coli*, *Enterobacter cloacae, and Klebsiella pneumoniae*, respectively.

Results were expressed by means of three independent experiments [14,41,42,43,44,45].

#### 4.2.3. Cytotoxicity Tests

The cytotoxicity of the raw extracts and fractions was evaluated using an MTT assay. This assay is based on the ability of the NAD(P)H-dependent cellular oxidoreductase enzyme present in the mitochondria of living cells to reduce MTT (yellow color) to its insoluble form Formazan (purple color). This color could be measured by a spectrophotometer, and the intensity of absorbance is proportional to the number of living cells [46]. IC_50_ (concentration that inhibits 50% of cell growth) values were determined as previously described [14,41,42,46].

#### 4.2.4. Parameters of the GC-MS

Gas chromatography-Electro ionization Mass Spectrometry (GC–EIMS) analysis was performed on a fused silica column (ZB-5-MS, 5% phenyl methyl polysiloxane, 30 m, 0.25 mm i.d., 0.25 µm film thickness; Phenomenex, Torrance, CA, USA) in a GC 2010 chromatograph coupled with GCMS-QP2010 SE mass spectrometer (Shimadzu, Kyoto, Japan) equipped with a quadrupole analyzer. Helium was used as a carrier gas at the flow rate of 1 mL/min. The programmed temperature was set as follows: oven temperature was raised from 100 °C to 325 °C using a ramp of 20 °C/min. The final temperature was maintained for 5 min (end of the analysis). One µL of the sample was injected into the column with a split ratio of 1/10. The MS detector was set as follows: electron impact mode (70 eV) with the ion source temperature set at 200°C, analyzed mass range *m/z* 40–700. Spectrum was acquired from 3 min (solvent delay) to 16.25 min (end of the run). The identification of the chemical structures was performed by comparison with a library of mass spectra (NIST MS Search 2.0).

## 5. Conclusions

Based on the most accurate information we have gathered on the plant species, our study is the first that has carried out in-depth research work on both the pharmacological activities (e.g., antimicrobial activity and cytotoxicity) and the phytochemical identification of the most active extracts or fractions of all parts (i.e., leaves, trunk barks, and roots) of *Daniellia oliveri*. Of note, all the parts of *D. oliveri* were active against the bacteria tested with MICs ranging from 16 to 256 µg/mL. Furthermore, whenever a MIC was not determined, inhibition of the bacterial growth was always reported at 256 µg/mL (the highest tested concentration) with values ranging from 15 to 90%. The most active part of the plant was the trunk barks, followed by the leaves and ending with the roots. Almost all the raw extracts and the fractions were active against Gram-positive bacteria (*E. faecalis* and *S. aureus*) except the dichloromethane fraction of the leaves, which were not active against *E. faecalis* at the highest tested concentration (i.e., 256 µg/mL). The GC-MS phytochemical investigation performed on the non-polar fractions of the leaves, trunk barks, and root extracts of *D. oliveri* revealed fatty acids with terpenes, sterols, and phenolic compounds. The trunk barks of *D. oliveri* are mainly composed of fatty acids, sterols, terpenes, and phenolic compounds. The root extracts, in contrast, were primarily comprised of terpenes, followed by fatty acids and phytosterols as the most abundant compounds. The observed activities of *D. oliveri* organs raw extracts and fractions confirm the traditional use of this plant to treat bacterial diseases. The results confirm the use of the plant in folk medicine in Togo for the treatment of infectious cutaneous diseases, namely intertrigo. As perspectives for future work, we have noticed during the chemical investigation that several compounds during the phytochemical investigation did not hit structures of compounds present in the library, although they present a very good resolved, gaussoid peak. Those compounds might be worthy of further chemical investigation by formal isolation and identification by NMR and or other mass spectrometric techniques. Some further work may consequently be needed to fully document the chemical composition of this plant by performing additional purification processes on extracts to obtain pure compounds. It will also be interesting to test the activity of the plant extracts against a wider range of Gram-positive bacteria (e.g., other bacterial species, including multidrug-resistant bacteria) as the results show a selective efficiency against those bacteria. Finally, this work, such as many others in the field, shows the importance of preserving nature and traditional knowledge.

## Figures and Tables

**Table 1:** Antibacterial and antifungal activities (i.e., MIC in µg/mL) of the raw extracts (MeOH 100%) of *Daniellia oliveri* parts.

	*E. faecalis*	*S. aureus*	*P. aeruginosa*	*A. baumannii*	*E. coli*	*K. pneumoniae*	*E. cloacae*	*C. albicans*
Leaves	**32**	**256**	>256 (*74%*)	>256 (*87%*)	>256 (*88%*)	>256 (*78%*)	>256 (*75%*)	>256
Bark	**16**	**128**	**256**	>256 (*84%*)	**256**	>256 (*75%*)	>256 (*86%*)	>256
Roots	**64**	**256**	>256 (*59%*)	>256 (*50%*)	>256 (*44%*)	>256 (*58%*)	>256 (*37%*)	>256

Raw extract: methanolic extract (MeOH 100%). Antibacterial activity is based on the determination of Minimum Inhibitory Concentration (MIC, i.e., concentration that inhibits 100% of bacterial growth) values. Bacterial strains: *Enterococcus faecalis* (ATCC 29212), *Staphylococcus aureus* (ATCC 29213), *Escherichia coli* (ATCC 25922), *Klebsiella pneumoniae* (ABC 42), *Enterobacter cloacae* (ABC 291), *Pseudomonas aeruginosa* (ATCC 27853) and *Acinetobacter baumannii* (ABC 14). Fungal strains: *Candida albicans*. MICs are marked in bold. >256: no MIC observed at the highest concentration (256 μg/mL) tested. (*X%*): PI (percentage of inhibition of bacterial growth) was observed at 256 μg/mL, the highest concentration under investigation. Each experiment has been repeated three times (n = 3).

**Table 2:** Antibacterial activities (i.e., MIC in µg/mL) of the fractions derived from the raw extract of *Daniellia oliveri*.

	Fraction	*E. faecalis*	*S. aureus*	*P. aeruginosa*	*A. baumannii*	*E. coli*	*K. pneumoniae*	*E. cloacae*
Leaves	Petroleum ether	**256**	**256**	**256**	**256**	**256**	>256	**256**
	Dichloromethane	>256	**256**	>256 (72%)	>256 (82%)	>256 (85%)	>256 (82%)	>256 (59%)
	Acetate	>256 (78%)	**256**	>256 (63%)	>256 (71%)	>256 (86%)	>256 (52%)	>256 (69%)
	Butanol	**64**	**128**	**128**	**256**	**256**	**256**	**256**
	Water	**64**	**256**	>256	>256 (83.5%)	**256**	>256 (63%)	>256 (90%)
Bark	Petroleum ether	**128**	**256**	>256	>256	>256 (74%)	**256**	>256 (46%)
	Dichloromethane	**256**	**256**	>256	>256 (67%)	>256 (71%)	>256 (67.24%)	**256**
	Acetate	**64**	**128**	>256 (79.5%)	**256**	**256**	>256 (78%)	>256 (76%)
	Butanol	**64**	**128**	**256**	**256**	>256 (82.5%)	>256 (70.5%)	**256**
	Water	**64**	**128**	**128**	>256 (85%)	**256**	**256**	**256**
Roots	Petroleum ether	**32**	>256 (70%)	>256 (75%)	>256 (20%)	>256 (68%)	>256 (19%)	>256 (39%)
	Dichloromethane	**32**	**128**	>256 (84%)	>256 (15%)	>256 (63%)	>256 (33%)	>256 (32%)
	Acetate	**64**	**64**	**128**	>256 (45%)	>256 (57%)	>256 (36%)	>256 (38.5%)
	MeOH remaining	**128**	**128**	>256 (74%)	>256 (79.5%)	>256 (55%)	>256 (75.5%)	>256 (38.5%)
ATB		**2**	**<1**	8	8	4	1	4

MICs are marked in bold. >256: no MIC observed at the highest concentration tested, 256 µg/mL, (*X%*): Percentage of inhibition (PI) at the highest concentration of extract tested (256 µg/mL). ATB: antibiotic use as a positive control (see Materials and Methods for details). Each experiment has been repeated three times (n = 3).

**Table 3:** Antibacterial activities (i.e., MIC in µg/mL) of the most effective fractions on different strains of *Staphylococcus* spp.

	Fraction	*S. aureus*		MRSA		*S. epidermidis*	
		MIC	PI_50_	MIC	PI_50_	MIC	PI_50_
Leaves	Petroleum ether	256	ND	>256	ND	>256	ND
	Butanol	128	1	**128**	1	**128**	1
	Water	256	1	**128**	1	**64**	1
Bark	Petroleum ether	256	ND	**256**	ND	**128**	ND
	Dichloromethane	256	ND	>256 *63.54%*	ND	**256**	ND
	Acetate	128	ND	**128**	ND	**256**	ND
	Butanol	128	1	**128**	1	**64**	1
	Water	128	1	**128**	1	**64**	1
Roots	Dichloromethane	128	ND	**128**	ND	**256**	ND
	Acetate	64	ND	>256	ND	>256	ND
	MeOH	128	ND	**64**	ND	>256	ND

MRSA: Methicillin-Resistant Staphylococcus aureus; S. epidermidis: Staphylococcus epidermidis. MICs are marked in bold. PI_50_: concentration at which 50% of bacterial growth is inhibited at a concentration lower than 256 µg/mL. ND: not determined. Each experiment has been repeated three times (n = 3).

**Table 4:** Toxicity (IC_50_ in µg/ mL) of the most effective fractions on MRC 5 cell lines with comparison to the raw extract (n = 3).

Plant Part	Extracts	IC_50_
Leaves	Methanol	>256
	Butanol	>256
	Water	>256
Bark	MeOH	>256
	Petroleum ether	198.02 ± 0.19
	Ethyl acetate	>256
	Butanol	81.18 ± 0.07
	Water	76.96 ± 0.06
Roots	MeOH	>256
	Dichloromethane	161.15 ± 0.18
	MeOH final	210.67 ± 0.23

>256: no toxicity was observed at the highest concentration tested (256 µg/mL). Each experiment has been repeated three times (n = 3).

**Table 5:** GC-MS results of dichloromethane fraction of *D. oliveri* roots.

n°	RT	RSI %	Area %	Name
1	5.27	94	15.2	δ-Cadinene
2	6.05	-	3.7	INH
3	9.19	-	4.9	INH
4	9.75	-	5.7	INH
5	9.93	96	70.5	Daniellic acid

Relative selectivity index (RSI: similarity of the spectrum with the structure according to the library), n°: Compound number, RT: Retention time in min, INH: compound did not hit a compound present in the library of mass spectra (NIST MS Search 2.0).

**Table 6:** GC-MS results of petroleum ether fraction of *D. oliveri* trunk bark.

n°	RT	RSI %	Area %	Name
1	3.53	-	0.6	INH
2	5.49	91	2.2	Mellein
3	5.74	-	0.59	INH (terpenoid)
4	5.90	-	0.85	INH (terpenoid)
5	7.59	95	1.67	Palmitic acid
6	8.45	92	3.31	Linoleic acid
7	8.54	86	0.49	Oleic acid
8	13.37	72	1.17	Stigmasterol
9	13.73	78	3.49	γ-sistosterol

Relative selectivity index (RSI: similarity of the spectrum with the structure according to the library), n°: Compound number, RT: Retention time in min, INH: compound did not hit a compound present in the library.

**Table 7:** GC-MS results of dichloromethane fraction of D. oliveri trunk bark after sylilation.

n°	RT	RSI %	Area %	Name
1	3.55	94	0.81	Glycerol
2	3.84	95	0.66	Succinic acid
3–4	4.07 *	78	0.26	Fumaric acid + INH
5	5.31	91	0.35	Vanillin
6–7	5.44 *	87–90	0.42	3-hydroxybenzoic acid + p-tyrosol
8	5.81	92	0.50	4-hydroxybenzoic acid
9	6.86	93	1.02	Protocatechuic acid
10	7.03	88	0.50	Myristic acid
11	7.27	87	0.48	Syringic acid
12	7.45	94	0.50	Palmitic acid (methyl ester)
13	7.53	93	1.42	*n*-Pentadecanoic acid
14	8.02	94	10.02	Palmitic acid
15	8.25	82	0.35	Ferrulic acid
16	8.30	92	0.35	Linoleic acid (methyl ester)
17	8.32	91	0.41	6-Octadecenoic acid (methyl ester).
18	8.36	76	0.89	9,12-Octadecadienoic acid (methyl ester)
19	8.71	87	0.46	Hexadecane-1,2-diol
20	8.81	95	9.44	Linoleic acid
21	8.83	94	10.23	Octadecenoic acid
22	8.94	96	2.97	Stearic acid
23	9.78	91	0.89	Arachidic acid
24	10.20	94	1.16	Gadoleic acid
25	10.32	94	0.69	Mono palmitin isomer
26	10.55	92	0.70	Behenic acid
27	10.97	89	1.06	Mono olein isomer
28	11.28	91	2.16	Lignoceric acid
29	11.41	77	0.90	epicatechin
30	11.52	79	0.35	catechin
31	11.82	-	0.66	INH
32	11.94	-	0.75	INH
33	11.65	85	0.99	Pentacosanoic acid
34	12.04	92	1.37	Hexacosanoic acid
35	12.21	-	0.68	INH
36	12.61	85	0.81	24-lignoceric acid
37	13.26	91	1.47	Campesterol
38	13.40	92	1.81	Stigmasterol
39	13.61	-	0.61	INH
40	13.77	93	5.42	β-sitosterol
41	14.52	88	1.61	Lupeol
42	14.56	91	10.79	β-Amyrin
43	14.69	85	1.35	Sitosterone

Relative selectivity index (RSI: similarity of the spectrum with the structure according to the library), n°: Compound number, RT: Retention time in min, INH: compound did not hit a compound present in the library. *: partial co-elution.

**Table 8:** Plant materials used in the study and extraction yields for each extract.

Plant	Part	Mass (kg) of Powder Material	Mass of Raw Extract Obtained	Mass of Raw Extract Preserved for Biological and Chemical tests	Mass of Ether Petroleum Fraction Obtained	Mass of DichloroMethane Fraction Obtained	Mass of Ethyl Acetate Fraction Obtained	Mass of Butanol Obtained	**Mass of Water Fraction Obtained**
*D. oliveri*	Leaves	3	419.8 g	4.6 g	6.7 g	33.4 g	7.1 g	99.9 g	165.5 g
	Stem barks	3	468.9 g	11.3 g	0.4 g	1.6 g	56.6 g	49.9 g	150.9 g
	Roots	3	126.9 g	4.8 g	3.5 g	8.7 g	1 g	-	101.5 g (final methanol fraction)

Raw extracts and fractions of the different parts of *Daniellia oliveri*.

## Data Availability

Not applicable.
